# Supplementary figures and images for: A comparison of the effectiveness of functional MRI analysis methods for pain research: The new normal
Source: PLoS One. 2020 Dec 14;15(12):e0243723. doi: 10.1371/journal.pone.0243723 (PMC7735591; doi:10.1371/journal.pone.0243723)

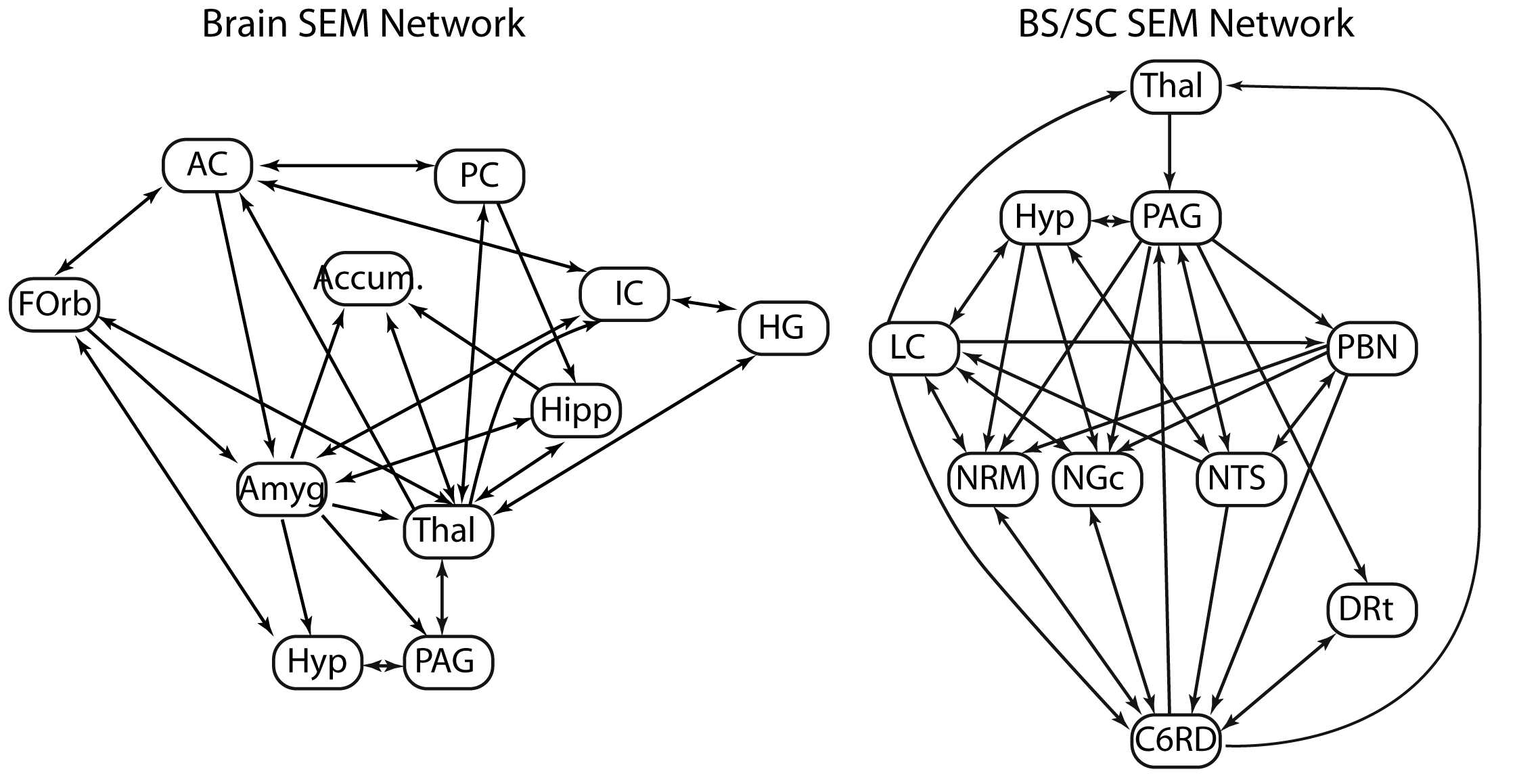

Supplement: S1 Fig — Abbreviations: AC: anterior cingulate cortex; Accum: nucleus accumbens; Amyg: amygdala; C6RD: right dorsal region of the 6th cervical spinal cord segment; DRt: dorsal reticular nucleus of the medulla; Forb: frontal orbital region; HG: heschel’s gyrus; Hipp: hippocampus; Hyp: hypothalamus; IC: insular cortex; LC: locus coeruleus; NGc: nucleus gigantocellularis; NRM: nucleus raphe magnus; NTS: nucleus tractus solitarius; PAG: periaqueductal gray region; PBN: parabrachial nucleus; PC: posterior cingulate cortex; Thal: thalamus. (TIF) [file pone.0243723.s001.tif]

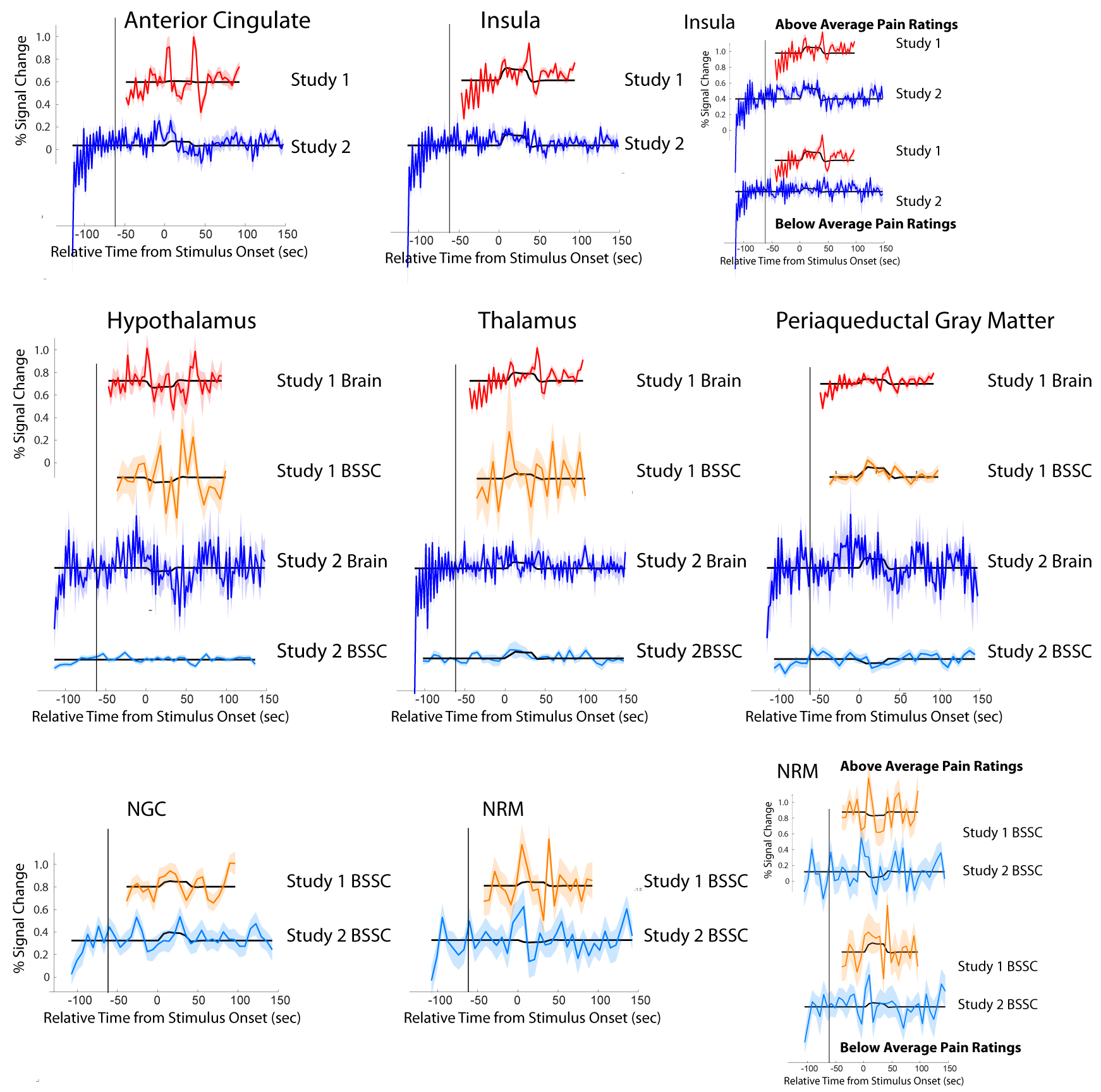

Supplement: S2 Fig — BOLD responses shown are averaged across all runs/participants in each study group, except where shown for pain ratings separated between above and below average values. The plots in each frame are offset vertically for clarity. The vertical axis indicates the relative scale for each frame. The horizontal axis indicates time, and plots are shown with the onset of stimulation aligned for each study. The vertical line indicates when participants were informed of which type of stimulation to expect in Study 2. The fit model paradigm to each time-series response is also shown in black on each plot. (TIF) [file pone.0243723.s002.tif]
