# Supplementary material for: A comparison of the effectiveness of functional MRI analysis methods for pain research: The new normal
Source: PLoS One. 2020 Dec 14;15(12):e0243723. doi: 10.1371/journal.pone.0243723 (PMC7735591; doi:10.1371/journal.pone.0243723)
Supplement: S1 Table — Abbreviations are listed in the caption for S1 Fig. (DOCX) [file pone.0243723.s003.docx]

**Study 1 and 2 Brain GLM**

| **Study 1** | | | | | **Study 2** | | | | |  |
| --- | --- | --- | --- | --- | --- | --- | --- | --- | --- | --- |
| **Region** | **T-value** | **MNI coords** | | | **Region** | **T-value** | **MNI coords** | | |  |
| AC | -4.68 | -6.3 | 37.5 | 9.8 | AC | -4.7 | -6.3 | 37.5 | 9.8 |  |
| HG | 4.55 | -15.4 | -18.6 | 7.9 | FOrb | -4.29 | -14.4 | 26.7 | -16.8 |  |
| Hypothalamus | -4.76 | -11.7 | -1.4 | -10.2 | FOrb | 4.49 | -10.1 | 26.4 | -17.8 |  |
| IC | 5.43 | 10.5 | 4.3 | 1.4 | IC | 3.77 | 12 | 5.3 | 3.3 |  |
| PAG | 4.16 | -6.7 | -28.5 | -10.1 | Thalamus | 3.66 | -8.9 | -17.6 | 7.6 |  |
| PC | -4.9 | -7 | -40.1 | 29.5 | Accumbens | -3.4 | -0.2 | 14.7 | -5.8 |  |
| Thalamus | 5.19 | -7.7 | -19.6 | 3.9 |  |  |  |  |  |  |
| Accumbens | -5.29 | -0.2 | 14.7 | -5.8 |  |  |  |  |  |  |
