# Supplementary material for: A comparison of the effectiveness of functional MRI analysis methods for pain research: The new normal
Source: PLoS One. 2020 Dec 14;15(12):e0243723. doi: 10.1371/journal.pone.0243723 (PMC7735591; doi:10.1371/journal.pone.0243723)
Supplement: S2 Table — The values shown are for the epoch spanning the stimulation period. Abbreviations are listed in the caption for S1 Fig. (DOCX) [file pone.0243723.s004.docx]

**Study 1 and 2 Brain Connectivity based on Correlation**

| **Study 1** | | | **Study 2** | | |
| --- | --- | --- | --- | --- | --- |
| **Region 1** | **Region 2** | **R** | **Region 1** | **Region 2** | **R** |
| AC | Amygdala | 0.52 | AC | FOrb | 0.38 |
| AC | FOrb | 0.63 | AC | HG | 0.33 |
| AC | HG | 0.63 | AC | Hippocampus | 0.31 |
| AC | Hippocampus | 0.66 | AC | IC | 0.55 |
| AC | IC | 0.71 | AC | PC | 0.51 |
| AC | PC | 0.76 | AC | Thalamus | 0.39 |
| AC | Thalamus | 0.64 | AC | Accumbens | 0.32 |
| AC | Accumbens | 0.49 | Amygdala | FOrb | 0.58 |
| Amygdala | FOrb | 0.47 | Amygdala | Hippocampus | 0.55 |
| Amygdala | HG | 0.41 | Amygdala | Hypothalamus | 0.32 |
| Amygdala | Hippocampus | 0.44 | Amygdala | IC | 0.37 |
| Amygdala | IC | 0.46 | FOrb | HG | 0.4 |
| Amygdala | NTS | 0.45 | FOrb | Hippocampus | 0.51 |
| Amygdala | PBN | 0.42 | FOrb | Hypothalamus | 0.32 |
| Amygdala | PC | 0.44 | FOrb | IC | 0.41 |
| Amygdala | Thalamus | 0.46 | FOrb | PAG | -0.47 |
| Amygdala | Accumbens | 0.47 | FOrb | Accumbens | 0.34 |
| FOrb | HG | 0.44 | HG | Hippocampus | 0.39 |
| FOrb | Hippocampus | 0.52 | HG | PC | 0.35 |
| FOrb | IC | 0.51 | HG | Thalamus | 0.32 |
| FOrb | PC | 0.55 | Hippocampus | Hypothalamus | 0.32 |
| FOrb | Thalamus | 0.42 | Hippocampus | IC | 0.46 |
| HG | Hippocampus | 0.55 | Hippocampus | PC | 0.47 |
| HG | IC | 0.7 | Hippocampus | Accumbens | 0.32 |
| HG | PC | 0.65 | Hypothalamus | IC | -0.36 |
| HG | Thalamus | 0.62 | IC | PAG | 0.63 |
| HG | Accumbens | 0.45 | IC | PC | 0.37 |
| Hippocampus | Hypothalamus | 0.43 | IC | Thalamus | 0.45 |
| Hippocampus | IC | 0.55 | PAG | Accumbens | 0.32 |
| Hippocampus | PC | 0.62 | PC | Thalamus | 0.36 |
| Hippocampus | Thalamus | 0.5 |  |  |  |
| Hippocampus | Accumbens | 0.42 |  |  |  |
| IC | PAG | 0.42 |  |  |  |
| IC | PC | 0.61 |  |  |  |
| IC | Thalamus | 0.72 |  |  |  |
| IC | Accumbens | 0.52 |  |  |  |
| PAG | Thalamus | 0.46 |  |  |  |
| PC | Thalamus | 0.57 |  |  |  |
| PC | Accumbens | 0.39 |  |  |  |
| Thalamus | Accumbens | 0.54 |  |  |  |
