# Supplementary material for: A comparison of the effectiveness of functional MRI analysis methods for pain research: The new normal
Source: PLoS One. 2020 Dec 14;15(12):e0243723. doi: 10.1371/journal.pone.0243723 (PMC7735591; doi:10.1371/journal.pone.0243723)
Supplement: S3 Table — The values shown are for the epoch spanning the stimulation period. Abbreviations are listed in the caption for S1 Fig. (DOCX) [file pone.0243723.s005.docx]

**Study 1 and 2 Brain SEM with 1 source**

| **Study 1** | | | **Study 2** | | |
| --- | --- | --- | --- | --- | --- |
| **Target** | **Source** | **β ± sem** | **Target** | **Source** | **β ± sem** |
| AC | FOrb | 0.37 ± 0.04 | AC | FOrb | 0.16 ± 0.02 |
| AC | IC | 0.98 ± 0.08 | AC | IC | 0.79 ± 0.07 |
| AC | PC | 0.82 ± 0.06 | AC | PC | 0.57 ± 0.07 |
| AC | Thalamus | 0.45 ± 0.04 | AC | Thalamus | 0.18 ± 0.04 |
| Amygdala | AC | 0.63 ± 0.12 | Amygdala | AC | 0.57 ± 0.13 |
| Amygdala | FOrb | 0.66 ± 0.11 | Amygdala | FOrb | 0.76 ± 0.07 |
| Amygdala | Hippocampus | 0.59 ± 0.12 | Amygdala | Hippocampus | 0.45 ± 0.05 |
| Amygdala | IC | 0.80 ± 0.14 | Amygdala | IC | 0.89 ± 0.14 |
| FOrb | AC | 0.87 ± 0.10 | FOrb | AC | 0.87 ± 0.13 |
| FOrb | Thalamus | 0.75 ± 0.14 | FOrb | Hypothalamus | 0.59 ± 0.11 |
| HG | IC | 0.94 ± 0.08 | FOrb | Thalamus | -1.45 ± 0.26 |
| HG | Thalamus | 0.58 ± 0.09 | HG | IC | 0.33 ± 0.07 |
| Hippocampus | Amygdala | 0.19 ± 0.04 | HG | Thalamus | 0.25 ± 0.05 |
| Hippocampus | PC | 0.43 ± 0.05 | Hippocampus | Amygdala | 0.41 ± 0.05 |
| Hippocampus | Thalamus | 0.55 ± 0.08 | Hippocampus | PC | 0.23 ± 0.05 |
| Hypothalamus | Amygdala | 0.48 ± 0.09 | Hippocampus | Thalamus | 0.19 ± 0.04 |
| IC | AC | 0.48 ± 0.05 | Hypothalamus | Amygdala | 0.57 ± 0.11 |
| IC | Amygdala | 0.19 ± 0.04 | Hypothalamus | FOrb | 0.16 ± 0.03 |
| IC | HG | 0.50 ± 0.04 | Hypothalamus | PAG | 0.36 ± 0.08 |
| IC | Thalamus | 0.64 ± 0.05 | IC | AC | 0.39 ± 0.04 |
| PAG | Thalamus | 0.74 ± 0.12 | IC | Amygdala | 0.15 ± 0.02 |
| PC | AC | 0.70 ± 0.05 | IC | HG | 0.23 ± 0.05 |
| PC | Thalamus | 1.03 ± 0.12 | IC | Thalamus | 0.18 ± 0.03 |
| Thalamus | Amygdala | 0.30 ± 0.04 | PAG | Hypothalamus | 0.18 ± 0.04 |
| Thalamus | FOrb | 0.22 ± 0.04 | PAG | Thalamus | 0.72 ± 0.17 |
| Thalamus | Hippocampus | 0.48 ± 0.07 | PC | AC | 0.38 ± 0.04 |
| Thalamus | PAG | 0.27 ± 0.04 | PC | Thalamus | 0.24 ± 0.04 |
| Thalamus | PC | 0.29 ± 0.03 | Thalamus | Amygdala | 0.18 ± 0.04 |
| Thalamus | Accumbens | 0.49 ± 0.06 | Thalamus | FOrb | -0.07 ± 0.01 |
| Accumbens | Amygdala | 0.25 ± 0.06 | Thalamus | Hippocampus | 0.51 ± 0.09 |
| Accumbens | Hippocampus | 0.67 ± 0.13 | Thalamus | PAG | 0.09 ± 0.02 |
| Accumbens | Thalamus | 0.59 ± 0.08 | Thalamus | PC | 0.47 ± 0.08 |
|  |  |  | Accumbens | Amygdala | 0.25 ± 0.06 |
|  |  |  | Accumbens | Hippocampus | 0.28 ± 0.07 |
