# Supplementary material for: A comparison of the effectiveness of functional MRI analysis methods for pain research: The new normal
Source: PLoS One. 2020 Dec 14;15(12):e0243723. doi: 10.1371/journal.pone.0243723 (PMC7735591; doi:10.1371/journal.pone.0243723)
Supplement: S4 Table — The values shown are for the epoch spanning the stimulation period. Abbreviations are listed in the caption for S1 Fig. (DOCX) [file pone.0243723.s006.docx]

**Study 1 and 2 Brain SEM with 2 Sources**

| **Study 2** | | | **Study 2** | | |
| --- | --- | --- | --- | --- | --- |
| **Target** | **Source** | **β ± sem** | **Target** | **Source** | **β ± sem** |
| AC | FOrb | 0.21 ± 0.04 | AC | FOrb | 0.16 ± 0.02 |
| AC | IC | 0.52 ± 0.08 | AC | IC | 0.66 ± 0.07 |
| AC | PC | 0.46 ± 0.05 | AC | PC | 0.42 ± 0.06 |
| AC | Thalamus | 0.22 ± 0.04 | AC | Thalamus | 0.15 ± 0.03 |
| Amygdala | Hippocampus | 0.28 ± 0.05 | Amygdala | FOrb | 0.52 ± 0.08 |
| HG | IC | 0.33 ± 0.06 | Amygdala | Hippocampus | 0.28 ± 0.03 |
| HG | Thalamus | 0.39 ± 0.08 | Amygdala | IC | 1.35 ± 0.20 |
| Hippocampus | Amygdala | 0.21 ± 0.04 | FOrb | AC | 2.53 ± 0.41 |
| Hippocampus | PC | 0.46 ± 0.06 | FOrb | Hypothalamus | 0.58 ± 0.10 |
| Hippocampus | Thalamus | 0.34 ± 0.07 | FOrb | Thalamus | -1.44 ± 0.25 |
| IC | AC | 0.31 ± 0.04 | Hippocampus | Amygdala | 0.67 ± 0.06 |
| IC | Amygdala | 0.13 ± 0.03 | Hippocampus | PC | 0.90 ± 0.19 |
| IC | HG | 0.26 ± 0.03 | Hippocampus | Thalamus | -0.68 ± 0.14 |
| IC | Thalamus | 0.43 ± 0.05 | Hypothalamus | FOrb | 1.36 ± 0.23 |
| PC | AC | 0.44 ± 0.08 | Hypothalamus | PAG | 0.43 ± 0.08 |
| PC | Thalamus | 0.29 ± 0.06 | IC | AC | 0.35 ± 0.03 |
| Thalamus | Amygdala | 0.21 ± 0.04 | IC | Amygdala | 0.11 ± 0.02 |
| Thalamus | FOrb | 0.29 ± 0.06 | IC | HG | 0.12 ± 0.02 |
| Thalamus | Hippocampus | 0.34 ± 0.07 | IC | Thalamus | 0.16 ± 0.03 |
| Thalamus | PAG | 0.17 ± 0.04 | PC | AC | 0.45 ± 0.05 |
| Thalamus | PC | 0.23 ± 0.03 | PC | Thalamus | 0.15 ± 0.03 |
| Thalamus | Accumbens | 0.37 ± 0.06 | Thalamus | Amygdala | -0.19 ± 0.04 |
|  |  |  | Thalamus | FOrb | -0.07 ± 0.01 |
|  |  |  | Thalamus | Hippocampus | 0.23 ± 0.05 |
|  |  |  | Thalamus | PC | 0.45 ± 0.08 |
