# Supplementary material for: A comparison of the effectiveness of functional MRI analysis methods for pain research: The new normal
Source: PLoS One. 2020 Dec 14;15(12):e0243723. doi: 10.1371/journal.pone.0243723 (PMC7735591; doi:10.1371/journal.pone.0243723)
Supplement: S5 Table — Data are from brain regions in Study 1. Values are listed for the epoch spanning the stimulation period. Abbreviations are listed in the caption for S1 Fig. (DOCX) [file pone.0243723.s007.docx]

**Study 1 and 2 Brain SEM with 2 sources, beta-values correlated with pain ratings**

| **Study 1** | | | **Study 2** | | |
| --- | --- | --- | --- | --- | --- |
| **Target** | **Source** | **Z** | **Target** | **Source** | **Z** |
| Amygdala | Thalamus | -3.7 | AC | IC | 3.9 |
| FOrb | AC | 3.7 | Amygdala | Hippocampus | 4.3 |
| FOrb | Amygdala | 3.3 | Amygdala | IC | 3.7 |
| FOrb | Thalamus | -3.5 | Amygdala | PAG | 4.3 |
| HG | IC | -3.5 | Amygdala | Accumbens | 3.4 |
| Hippocampus | Amygdala | 3.7 | FOrb | AC | 4 |
| Hypothalamus | FOrb | -3.2 | FOrb | Hypothalamus | -3.4 |
| Hypothalamus | PAG | 3.4 | Hippocampus | Amygdala | 3.9 |
| IC | Amygdala | 3 | Hippocampus | Thalamus | -3.5 |
| PAG | Hypothalamus | 3.3 | Hippocampus | Accumbens | 5 |
| PAG | Thalamus | 3.7 | Hypothalamus | FOrb | 3.4 |
| PC | Thalamus | 3 | Hypothalamus | PAG | 4 |
| Thalamus | FOrb | -3.4 | IC | AC | 4.6 |
| Thalamus | HG | -3.4 | PAG | Thalamus | 3.7 |
| Thalamus | PAG | 3 | PC | Hippocampus | 3.7 |
| Thalamus | Accumbens | 3.2 |  |  |  |
| PC | AC | 3.3 |  |  |  |
| PC | Hippocampus | 3.6 |  |  |  |
| PC | Thalamus | 4.1 |  |  |  |
| Thalamus | AC | -3.1 |  |  |  |
| Thalamus | FOrb | -3.6 |  |  |  |
| Thalamus | Hippocampus | -3.8 |  |  |  |
| Thalamus | PAG | 3.3 |  |  |  |
| Thalamus | PC | -3.2 |  |  |  |
| Thalamus | Accumbens | 3.1 |  |  |  |
| Accumbens | Thalamus | -4.5 |  |  |  |
