# Supplementary material for: A comparison of the effectiveness of functional MRI analysis methods for pain research: The new normal
Source: PLoS One. 2020 Dec 14;15(12):e0243723. doi: 10.1371/journal.pone.0243723 (PMC7735591; doi:10.1371/journal.pone.0243723)
Supplement: S6 Table — Abbreviations are listed in the caption for S1 Fig. (DOCX) [file pone.0243723.s008.docx]

**Study 1 and 2 BS/SC GLM**

| **Study 1** | | | **Study 2** | | | | |
| --- | --- | --- | --- | --- | --- | --- | --- |
| **Region** | **T-value** | **MNI coords** | **Region** | **T-value** | **MNI coords** | | |
|  |  |  | C6RD | -4.14 | 2.7 | -46.6 | -99.3 |
|  |  |  | NRM | -3.6 | 1.5 | -36 | -53.9 |
