# Supplementary material for: A comparison of the effectiveness of functional MRI analysis methods for pain research: The new normal
Source: PLoS One. 2020 Dec 14;15(12):e0243723. doi: 10.1371/journal.pone.0243723 (PMC7735591; doi:10.1371/journal.pone.0243723)
Supplement: S7 Table — Values are listed for the epoch spanning the stimulation period. Abbreviations are listed in the caption for S1 Fig. (DOCX) [file pone.0243723.s009.docx]

**Study 1 and 2 BS/SC Connectivity based on Correlation**

| **Study 1** | | | **Study 2** | | |
| --- | --- | --- | --- | --- | --- |
| **Region 1** | **Region 2** | **R** | **Region 1** | **Region 2** | **R** |
| Hypothalamus | LC | 0.57 | Hypothalamus | Thalamus | 0.47 |
| Hypothalamus | PAG | 0.46 |  |  |  |
| Hypothalamus | PBN | 0.44 |  |  |  |
| Hypothalamus | Thalamus | 0.8 |  |  |  |
| LC | PAG | 0.5 |  |  |  |
| LC | Thalamus | 0.59 |  |  |  |
| NGC | NTS | 0.44 |  |  |  |
| PAG | Thalamus | 0.58 |  |  |  |
| PBN | Thalamus | 0.45 |  |  |  |
