# Supplementary material for: A comparison of the effectiveness of functional MRI analysis methods for pain research: The new normal
Source: PLoS One. 2020 Dec 14;15(12):e0243723. doi: 10.1371/journal.pone.0243723 (PMC7735591; doi:10.1371/journal.pone.0243723)
Supplement: S8 Table — Values are listed for the epoch spanning the stimulation period. Abbreviations are listed in the caption for S1 Fig. (DOCX) [file pone.0243723.s010.docx]

**Study 1 and 2 BS/SC SEM with 1 Source**

| **Study 1** | | | **Study 2** | | |
| --- | --- | --- | --- | --- | --- |
| **Target** | **Source** | **β ± sem** | **Target** | **Source** | **β ± sem** |
| C6RD | LC | 0.38 ± 0.07 | Hypothalamus | PAG | 0.17 ± 0.04 |
| C6RD | NGC | 0.32 ± 0.07 | LC | PAG | 0.24 ± 0.04 |
| C6RD | PBN | 0.33 ± 0.07 | PAG | Hypothalamus | 0.20 ± 0.05 |
| DRt | PAG | 0.22 ± 0.05 | PAG | Thalamus | 0.68 ± 0.13 |
| Hypothalamus | LC | 0.39 ± 0.04 | PBN | LC | 0.20 ± 0.01 |
| Hypothalamus | NTS | 0.16 ± 0.03 | PBN | PAG | 0.17 ± 0.04 |
| Hypothalamus | PAG | 0.44 ± 0.04 | Thalamus | LC | 0.19 ± 0.01 |
| LC | Hypothalamus | 0.41 ± 0.04 |  |  |  |
| LC | NTS | 0.20 ± 0.04 |  |  |  |
| LC | PAG | 0.52 ± 0.04 |  |  |  |
| NGC | C6RD | 0.10 ± 0.02 |  |  |  |
| NGC | Hypothalamus | 0.24 ± 0.04 |  |  |  |
| NGC | LC | 0.17 ± 0.04 |  |  |  |
| NGC | PAG | 0.25 ± 0.05 |  |  |  |
| NGC | PBN | -0.07 ± 0.02 |  |  |  |
| NRM | PAG | 0.25 ± 0.06 |  |  |  |
| NTS | Hypothalamus | 0.27 ± 0.05 |  |  |  |
| NTS | PAG | 0.19 ± 0.04 |  |  |  |
| NTS | PBN | 0.20 ± 0.04 |  |  |  |
| PAG | C6RD | 0.15 ± 0.02 |  |  |  |
| PAG | Hypothalamus | 0.43 ± 0.04 |  |  |  |
| PAG | NTS | 0.16 ± 0.04 |  |  |  |
| PAG | Thalamus | 0.87 ± 0.05 |  |  |  |
| PBN | LC | 0.38 ± 0.05 |  |  |  |
| PBN | NTS | 0.16 ± 0.03 |  |  |  |
| PBN | PAG | 0.31 ± 0.04 |  |  |  |
| Thalamus | C6RD | 0.10 ± 0.01 |  |  |  |
| Thalamus | LC | 0.35 ± 0.02 |  |  |  |
