# Supplementary material for: A comparison of the effectiveness of functional MRI analysis methods for pain research: The new normal
Source: PLoS One. 2020 Dec 14;15(12):e0243723. doi: 10.1371/journal.pone.0243723 (PMC7735591; doi:10.1371/journal.pone.0243723)
Supplement: S9 Table — Abbreviations are listed in the caption for S1 Fig. (DOCX) [file pone.0243723.s011.docx]

**Study 1 BS/SC SEM with 2 Sources**

| **Study 1** | | | **Study 2** | | |
| --- | --- | --- | --- | --- | --- |
| **Target** | **Source** | **β ± sem** | **Target** | **Source** | **β ± sem** |
| Hypothalamus | LC | 0.23 ± 0.03 |  |  |  |
| Hypothalamus | NTS | 0.15 ± 0.03 |  |  |  |
| Hypothalamus | PAG | 0.33 ± 0.04 |  |  |  |
| LC | Hypothalamus | 0.23 ± 0.04 |  |  |  |
| LC | NTS | 0.20 ± 0.04 |  |  |  |
| LC | PAG | 0.42 ± 0.04 |  |  |  |
| NGC | LC | 0.08 ± 0.01 |  |  |  |
| NGC | PAG | 0.24 ± 0.05 |  |  |  |
| PAG | C6RD | 0.10 ± 0.02 |  |  |  |
| PAG | Hypothalamus | -0.30 ± 0.06 |  |  |  |
| PAG | NTS | 0.19 ± 0.04 |  |  |  |
| PAG | Thalamus | 1.09 ± 0.06 |  |  |  |
| PBN | LC | 0.31 ± 0.05 |  |  |  |
| PBN | NTS | -0.65 ± 0.10 |  |  |  |
| PBN | PAG | 0.27 ± 0.04 |  |  |  |
| Thalamus | C6RD | 0.06 ± 0.01 |  |  |  |
| Thalamus | LC | 0.32 ± 0.02 |  |  |  |
