# Supplementary material for: A comparison of the effectiveness of functional MRI analysis methods for pain research: The new normal
Source: PLoS One. 2020 Dec 14;15(12):e0243723. doi: 10.1371/journal.pone.0243723 (PMC7735591; doi:10.1371/journal.pone.0243723)
Supplement: S10 Table — Data are from brainstem and spinal cord regions in Studies 1 and 2. The Z-score is computed from the correlation, R, value, and reflects the significance of the correlation. Only significant values are shown (corrected for multiple comparisons, etc, etc). Values are listed for the epoch spanning the stimulation period. Abbreviations are listed in the caption for S1 Fig. (DOCX) [file pone.0243723.s012.docx]

**Study 1 and 2 BS/SC SEM with 2 sources, beta-values correlated with pain ratings**

| **Study 1** | | | **Study 2** | | |
| --- | --- | --- | --- | --- | --- |
| **Target** | **Source** | **Z** | **Target** | **Source** | **Z** |
| C6RD | NGC | -3.3 | C6RD | DRt | 3.2 |
| C6RD | PAG | -3.1 | C6RD | NGC | 3.1 |
| Hypothalamus | NGC | 3.1 | DRt | C6RD | 3.1 |
| Hypothalamus | PAG | -3.4 | Hypothalamus | PAG | -3.4 |
| LC | C6RD | -3.2 | LC | NRM | -3.1 |
| LC | NRM | 3 | NGC | C6RD | -3.2 |
| NGC | C6RD | -3.3 | NRM | C6RD | -3 |
| PAG | LC | -3.4 | NRM | LC | -3.3 |
|  |  |  | NTS | PAG | -3 |
|  |  |  | PAG | LC | -3.3 |
|  |  |  | PAG | NGC | -5.3 |
|  |  |  | PBN | NRM | -3 |
|  |  |  | PBN | NTS | 3.1 |
