# Supplementary material for: A comparison of the effectiveness of functional MRI analysis methods for pain research: The new normal
Source: PLoS One. 2020 Dec 14;15(12):e0243723. doi: 10.1371/journal.pone.0243723 (PMC7735591; doi:10.1371/journal.pone.0243723)
Supplement: Attachment — Submitted filename: PLOSone responses to reviewers comments Nov_02_2020.docx [file pone.0243723.s013.docx]

**PLOSone revisions: PONE-D-20-23531**

We appreciate the time taken by the reviewers and their helpful comments. We have revised the manuscript according to each of the editor’s comments, and the comments from each of the reviewers as detailed below. For clarity we have repeated the comments (in italics), followed by our responses. When possible, without losing context, we have shortened the comments. We believe that the revisions have improved the quality of the manuscript.

**Editor’s comments:**

*Journal Requirements:*

*When submitting your revision, we need you to address these additional requirements.*

*1. Please ensure that your manuscript meets PLOS ONE's style requirements, including those for file naming. The PLOS ONE style templates can be found at …*

We have revised the file names, figure styles, etc., to conform with PLOSone styles.

*2. We note that you have stated that you will provide repository information for your data at acceptance. Should your manuscript be accepted for publication, we will hold it until you provide the relevant accession numbers or DOIs necessary to access your data. If you wish to make changes to your Data Availability statement, please describe these changes in your cover letter and we will update your Data Availability statement to reflect the information you provide.*

Once the data uploaded to FigShare are made public, the DOI will be 10.6084/m9.figshare.13176860 .

*3. Please clarify in your Ethics statement if the authors of this study had access to identifying information. Please also add this information, along with the rest of the Ethics statement, to your Methods section.*

The ethics statement has been revised to clarify that data were anonymized. The ethics statement has also been included in the Methods section, as follows:

“The studies from which data were obtained were approved by the Health Sciences

Research Ethics board at Queen's University. All participants provided informed written

consent, prior to participating.”

**Reviewers' comments:**

***Reviewer #1:*** *Stroman et al. present a manuscript of the impact of different fMRI analyses on pain research, describing model-driven and data-driven methods and respective findings. This is a very interesting manuscript that shows how analyses in the neuroimaging field can be useful to investigate mechanisms related to pain. The manuscript is well written and the discussion satisfactorily show the most effective method for fMRI studies of pain. However, I still have some suggestions to improve the manuscript. I remain enthusiastic about the goal of this study and the discussion made by the authors.*

*1. Methods – Brain fMRI data: The authors stated: “The data were converted to NIfTI format, motion and slice time corrected, co-registered to their anatomical images, and normalized to the MNI template (Montreal Neurological Institute, Montreal, Quebec).” Please describe the preprocessing steps in more detail, including quality control methods to account for motion.*

We have added details to the Methods section as suggested:

The data were converted from DICOM to NIfTI format, and were then co-registered to the third volume in the time-series to correct for motion, and motion parameters were retained for later noise modeling. We considered motion exceeding one voxel width, or 5 degrees of rotation, to be excessive, and no data sets were discarded because of excess motion. Slice-timing correction was applied by interpolating to the middle of each volume acquisition period. Spatial normalization was then applied to the data from each participant by first normalizing the fMRI data to the anatomical images from the same participant, to make use of the better data quality of the anatomical images, and then normalizing the anatomical images to the MNI template. The complete transformation needed to map the fMRI data to the MNI template (Montreal Neurological Institute, Montreal, Quebec) was thus determined.

*2. Methods – Study 2: Please mention the body location of Study 2’s stimulation.*

This detail has been added to the text.

*3. Methods: Please describe the methods and software used to create figures 2, 3 and 4.*

These details have been added, as follows:

Methods Section, “Definition of Clusters” subsection:

The regions used are displayed in Figure 2 by generating a 3D plot in MATLAB, rendering the region surfaces using the MATLAB functions “isosurface”, “patch”, and “isonormals”, and assigning each region a different colour. The 2D figures are then captured in PDF format from the 3D plots and paste into Adobe Illustrator for adding text annotations and colour bars.

and

Results section (referring to Fig 3 and 4):

These figures were generated using the methods described above for Figure 2, with the addition of significant connections between regions indicated with lines plotted in MATLAB.

*4. Discussion: Even though, the study had a consistent sample, i.e. only women, the age differences were significant. Please add some sentences to the discussion pointing out the potential effects of age and also sex on these findings. There is a vast literature discussing sex and age differences in brain regions involved in pain processing.*

Respectfully, the age ranges were not significantly different between the two study groups. Participants in Study 1 had an age range of 21-55 years, average of 39.1 ± 10.2 years (mean ± std), and participants in Study 2 had an age range of 21-59, average 36 ± 11.3 years. While age and sex differences are certainly important effects for studies of pain, the focus of this study is to compare the performance of analysis methods for fMRI studies. We do not have any evidence to suggest that our findings would be different in groups of males instead of females, or in groups of participants with different ages. We understand the reviewer’s point however, and we have added to the Discussion as follows: “This comparison of methods included data from females only, with average ages of 39 years and 36 years for the two study groups, and we did not investigate the effects of sex-differences on pain, or the effects of age, which could introduce even greater variability across participants.”

**Reviewer #2**:

*Thank you for providing me the opportunity to review the manuscript by Stroman et al comparing the effectiveness of fMRI analysis in the context of brain research. Firstly, I would like to congratulate the authors on a very important and well-designed study, well-written manuscript. Please find below some comments:

• The introduction is lengthy and thus, the authors are encouraged to shorten it. Parts of the introduction can be moved to the discussion.*

While we understand the reviewer’s point, that the Introduction does contain quite a lot of information, it was originally only 621 words. This is not particularly long for an introduction to a paper. We feel that the points raised in the Introduction are essential for providing background to explain the rationale for our study and the approach taken, and to illustrate the importance of doing this study. Nonetheless, we have revised the introduction in an effort to make it easier to read, and to shorten it, but we were only able to decrease the length to 569 words. We believe that the points made are essential background for the remainder of the paper.

*• Page 3, line 75: Spinal cord and brainstem can be abbreviated as the authors have introduced these abbreviations earlier in the introduction.*

These changes have been made in the text.

*• Is there any overlap in the study participants of study 1 and 2? I.e., Are all participants unique or did some take part in both studies?*

There was no overlap that we are aware of between the two studies. We specified that the data were collected at least 4 years apart (2013-2014 for Study 1 and 2018-2019 for Study 2), and we have now clarified that the data were anonymized prior to being used for the present study.

*• What was the rationale to only include female subjects?*

The data were available from prior studies of pain processing in females, and included both BS/SC and brain fMRI data. In many cases participants returned for two visits and we were able to collect data from both regions in the same participants. We included only female subjects primarily because these data sets were available, and secondarily because they provided somewhat homogeneous groups that could be used for comparing the methods in this study.

*• Can the authors provide a table with the individual data for both cohorts? Please add the information regarding assess anxiety, depression, pain catastrophizing, social desirability, and health-related factors. Even if it was not used for the analysis. This table can go to the supplementary material if needed.*

Respectfully, we feel that this information is not relevant to the present study which has the focus of comparing analysis methods. The results we discuss are not related to pain processing specifically, or how it is influenced by personal characteristics. The methods we compare are applied to all of the data sets equivalently. If factors such as anxiety, depression, etc, influenced a participant’s pain responses, then the influence was the same for all of the analysis methods. While in the original studies the participants were characterized with questionnaires, we did not use these data for the comparison of methods. In the data shared on FigShare, we include the participants’ pain ratings, stimulation temperatures, and ages.

*• Did the authors assess handedness in their participants? The authors state, that the thermode was placed on the right hand. Was this the dominant hand for everyone?*

We did not assess handedness. It was important for the two previous studies that we stimulated the same side of the body, in order to evoke activity in the same regions of the CNS. It was also important that we assessed the pain responses, and observed the corresponding BOLD responses, for the stimulus that was applied, at the time of the study. If handedness affected the results, then it would be reflected in both the BOLD responses and the corresponding behavioral responses.

For the present study, it is important that we applied the analysis methods that we are comparing to all data sets, in order to compare the performance of the methods. If handedness affected the results, then it would have the same effect on all analysis methods, and we can generalize our findings to any fMRI study.

*• The authors state: ‘Only the data from the TSSP condition are used for the present comparison of methods’. What was the rationale to exclude TSSP-C?*

The TSSP-C condition was only in Study 1. The control condition in Study 2 did not include a stimulation period. As a result, we cannot directly compare the control conditions from the two studies, and so these data sets were not used.

*• How did the authors correct for multiple comparisons? What method was applied (e.g., Bonferroni)?*

In the Methods section the method is described as:

“Statistical thresholds used to infer significance were corrected for multiple comparisons, in every case. In order to confirm that suitable thresholds were used, all analysis methods were applied to replicated data sets consisting of random values, to create “null” data sets in which no significant values should be detected. Statistical distributions were thus determined, and statistical thresholds were identified to ensure that there was less than a 5% chance of a single false-positive (Type I) result occurring in any analysis (i.e. family-wise-error corrected p < 0.05).”

The correction for multiple comparisons is thus equivalent to a Bonferroni correction (i.e. limited to a 5% chance of a single false-positive occurring in any analysis), with the correction being applied to the actual number of independent comparisons in our analyses.

*• The authors are encouraged to make the code for analysis openly accessible. This would allow researchers to fully understand the analysis conducted, including the small details.*

Key components of the software used for the analyses are freely shared. The SPM12 software used for processing the brain fMRI data is available from the Wellcome Institute of Cognitive Neurology, London, UK, and the SpinalfMRI9 software used for brainstem/spinal cord data was developed by our lab, and is freely shared. This software includes the anatomical templates and region maps, and the methods for structural equation modeling (SEM) of any data sets (brain or BS/SC).

The remaining analyses that are described consist of connectivity analyses by means of temporal correlations, determining the amount of variance that is accounted for with connectivity analyses (SEM or correlations) and computing correlations between connectivity values and pain ratings. We have fully described the key aspects of the analysis methods in the manuscript, so that our findings can be replicated. We will make our data sets and participant characteristics available on FigShare if this manuscript is accepted for publication.

*• Even though details on the study participants are described in previous studies, it might be useful for the reader if there is a summary table about the participants in the supplementary material.*

In response to a similar comment above we have explained why we have not included a table of scores of anxiety, depression, etc. In the manuscript we described the participant demographics in terms of age and sex. The information we have provided is focussed on the pain stimulus and stimulation paradigm, and how the data were collected and analyzed. The focus of this paper is on the analysis methods that we are comparing, as opposed to investigating the pain responses or how they compare across groups/participants. It is important that we maintain the focus of this paper. However, as we specified above, we have included the participant characteristics (pain ratings, stimulation temperatures, ages) with the data that are shared in FigShare.

*• Figure 5: The authors show the R^2 values of their analysis. However, the bar plot is not an ideal choice to visualize these results as it implies a range. Thus, the authors are encouraged to use a dotplot or similar.*

Respectfully, bar plots do not imply a range. Perhaps the reviewer is thinking of whisker plots.

We feel that the bar plot is the clearest way to demonstrate the differences between the performance of the analysis methods that we tested. The differences are easy to see at a glance, and the large area covered by the bars is easy to identify with different colours. We believe that the plots are highly effective at conveying our results.

*• In the supplementary tables, please specify the abbreviations (even though they are explained in the main text) as these are ‘standalone’ objects that should be understandable without the main text.*

The abbreviations have been added to the figure and table captions.

*• The authors show the results from two cross-sectional studies. What is the authors experience in the reliability of such measurements within subjects at different timepoints? Please comment on that.*

The data used in this comparison of methods were from studies involving two visits on different days, but in one visit fMRI data were acquired from the BS/SC, and in the other visit fMRI data were acquired from the brain. As a result, we are not able to compare measurements at two different time points. Also, some participants did not return for two visits.

However, our previous studies have demonstrated that our measurements are quite reliable, given that we are able to detect differences in fMRI results relating to differences in pain responses between people, and that we can detect differences in responses related to changes in mood, attention focus, expectation of different stimuli, etc. These studies are mentioned in the Introduction to the manuscript.

*• Also, this study focuses on healthy controls that undergo experimental pain conditions. What is the authors opinion on the generalizability of this data-driven ad hypothesis driven methods, when applied to clinical pain populations? Also in the context of pain population undergoing experimental pain paradigms.*

We believe that the findings of this comparison are generalizable to all fMRI studies of pain. The differences in performance of the analysis methods that we compared were consistent across regions (BS/SC and brain) and were reproducible (Study 1 and Study 2). The differences in performance do not appear to depend on specific features of pain responses, and we have shown in previous papers as well that there is a continuous component of pain regulation that appears to be a general feature across pain studies. The present manuscript demonstrates that this continuous component is not represented by fitting to a model based on the timing of peripheral stimulation, and that analysis methods need to be adapted, such as by using data-driven methods.

We have carried out previous studies of participants with fibromyalgia, with experimental pain paradigms, and have found that data-driven methods are more effective in this clinical population. For the present study it was important that we focus on the methods, and not confound the comparison by introducing a clinical population. We are continuing to develop the methods and are applying them to clinical populations, and these findings will be reported in future manuscripts.

*• Please add a link to the figshare repository, where the data is stored and publicly available, in the manuscript.*

The data have been uploaded to FigShare and the data will become publically available if this paper is accepted. Once it is made public the DOI will be 10.6084/m9.figshare.13176860
